# Bilateral visual loss as the initial presentation of chronic myeloid leukemia: a case report

**DOI:** 10.1097/MS9.0000000000002570

**Published:** 2024-09-12

**Authors:** Salah Marwan Saadeldine, Yara Mohammad Alammouri

**Affiliations:** a Department of Internal Medicine (Oncology), Faculty of Medicine, Damascus University, AL fayhaa Hospital; b Department of Internal Medicine (Neurology), Faculty of Medicine, Damascus University, AL fayhaa Hospital, Damascus, Syrian Arab Republic

**Keywords:** acute myeloid leukemia, case report, chronic myeloid leukemia, hematopoietic transplantation, visual loss

## Abstract

**Introduction:**

Chronic myeloid leukemia (CML) is the most common leukemia in adults. It can present with a wide variable range of symptoms and signs related to the phase of the disease. Ophthalmic manifestations as the first presentation of CML are unique, although they can occur during any stage of the disease. Ocular lesions in CML patients may be asymptomatic, so all patients should undergo an eye evaluation at the initial diagnosis.

**Case presentation:**

The authors report a case of a 17-year-old Syrian male who initially presented with progressive loss of vision, fatigue, and abdominal pain. Clinical examination showed bilateral retinal aneurysm hemorrhage, jaundice, and splenomegaly. Bone marrow biopsy results were consistent with the diagnosis of CML returning to AML. The patient was treated with intensive chemotherapy and then prepared for hematopoietic transplantation.

**Discussion:**

CML can present with variable symptoms and signs, but the ophthalmic manifestations are uncommon. Ophthalmic problems occur either from infiltration of neoplastic cells or from secondary causes, like thrombocytopenia, leukocytosis, hyperviscosity syndrome, or leukoembolization. In the literature, only some case reports presented eye involvement in CML as the first manifestation.

**Conclusion:**

Although this is a rare presentation of CML, we believe that it should be taken into consideration when managing these situations to obtain the right diagnosis and better treatment results. Collaboration between hematologists and ophthalmologists is necessary in deciding the treatment. Acute myeloid leukemia needs immediate medical attention and different treatment from CML.

## Introduction

Highlights

- Ophthalmic manifestations as the first and the only presentation of CML in patients are very rare.
- The challenges in our case are that the patient presented with ocular injury at the initial diagnosis, transformed to acute myeloid leukemia immediately.
- Collaboration between hematologists and ophthalmologists is necessary in deciding on treatment.

Chronic myeloid leukemia (CML) accounts for ~20% of leukemias in adults and is associated with a reciprocal chromosomal translocation t (9; 22) (q34; q11) resulting in a BCR-ABL fusion gene (Philadelphia chromosome)^1^. This translocation results in a new hybrid protein (BCR-ABL) with overactive tyrosine kinase activity and is implicated in the development of CML. Approximately 95% of patients with CML have this chromosome, which has been the target for drug design and treatment of this disorder^2^. CML can present with a wide range of symptoms and signs and can be diagnosed as an incidental finding on a routine complete blood count (CBC)^3^. CML is divided into three phases, based mainly on the number of immature white blood cells (blasts) in the blood or bone marrow. The three phases are chronic phase, accelerated phase, and blast phase (also called acute phase or blast crisis)^4^. Symptoms of CML include fever, loss of weight, and night sweats; however, splenomegaly remains the most common initial presentation in the chronic phase of the disease. With progressive disease, the accelerated or blast phase of CML can present similarly to acute leukemia, with signs and symptoms of anemia, bleeding, petechiae, ecchymosis, fever, or arthralgia. Rarely CML can also turn into acute leukemia, which needs immediate medical attention acute myeloblastic leukemia (AML) with BCR/ABL+ is considered to carry a worse prognosis, and hence its management approach is different from CML. However, AML with BCR/ABL+ was included as a separate provisional entity by the WHO classification of myeloid neoplasms in 2016^3–5^.

However, the ophthalmic manifestations are uncommon presentations of CML, can be variably described as blurred vision or even severe visual loss, or can be found coincidentally during a routine eye examination. The ocular manifestations may be the initial signs of leukemia or can happen during relapses. These ocular complications may result directly from metastatic leukemic infiltrates or are associated with thrombocytopenia, leukocytosis, hyperviscosity syndrome, or leukoembolization. Almost any ocular structure can be involved, and manifestations can vary from mild dot and blot hemorrhages to advanced optic nerve infiltration.^3–5^

We presented a rare case of retinopathy secondary to CML, which transformed to AML.

## Case presentation

**Figure 1:**
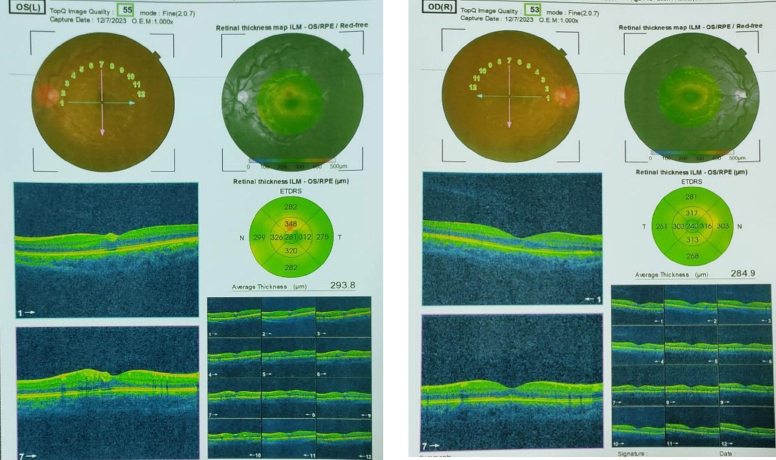
The optical coherence tomography revealed bilateral retinal blot aneurysm hemorrhages and retinal hemorrhage over both.

A 17-year-old male attended our hospital for a hematology consultation in December 2023. He presented with sudden loss of vision in both his eyes, general fatigue, weakness, arthralgia, and left upper quadrant abdominal pain. He was diagnosed with Gilbert syndrome in childhood with a family history of Gilbert syndrome with no history of neoplasm. Clinical examination showed jaundice, pallor, and splenomegaly (~15 cm below the left costal margin) with no hepatomegaly and no lymphopathy. The patient had visual acuity reduced to 2/20 bilaterally. Fundoscopic examination and optical coherence tomography (OCT) revealed bilateral retinal blot aneurysm hemorrhages and retinal hemorrhage over both foveae (Fig. 1). Blood test results showed anemia with thrombocytopenia and leukocytosis, high uric acid level, and high lactate dehydrogenase level. They are shown in the (Table 1). Computed tomographic scan (CT) and ultrasonography revealed splenomegaly 27 cm. Brain MRI and magnetic resonance angiography (MRA) findings were normal for the brain, and the intracranial arterial circle of Willis with no aneurysm or other vascular malformation was demonstrated.

**Table 1:** Blood test results.

WBCs (×10^3^/ml)	378	RBC (×10^6^/ml)	2.9	ESR (mm/h)	30
Neutro%	81%	Hb (g/l)	8.8	LDH (U/l)	880
Lympho%	3%	Hct	25	Uric acid (mg/dl)	11.3
Mono%	4%	MCV (fl)	80	Ca (mg/dl)	9.2
Eosino%	2%	PLTs (×10^3^/ml)	99	Na (mEq/l)	138
K (mEq/l)	3.2				

ESR, erythrocyte sedimentation rate; Hb, hemoglobin; Hct, hematocrit; Plt, platelet; LDH, lactate dehydrogenase; MCV, mean corpuscular volume; RBC, red blood cell; WBC, white blood cell.

**Figure 2:**
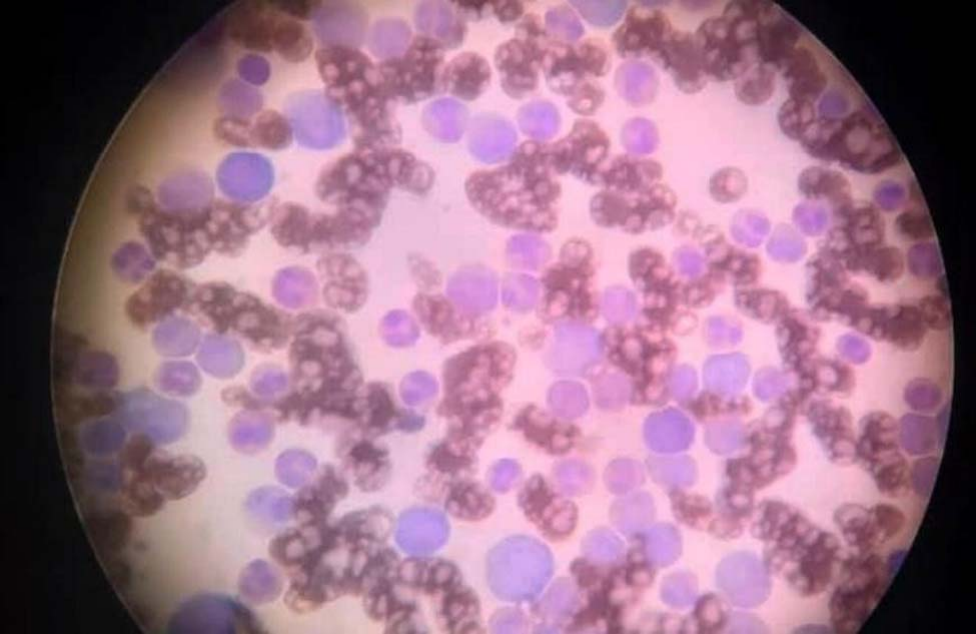
The peripheral blood smear showed variations in size and shape of red blood cells with highly elevated white blood cells left-shifted with some myeloblast and thrombocytopenia.

The peripheral blood smear showed variations in size and shape of red blood cells with highly elevated white blood cells left-shifted with some myeloblast and thrombocytopenia (Fig. 2). These findings may be consistent with the CML so we obtained a bone marrow aspiration and biopsy. The bone marrow aspiration was suggestive of myeloproliferative neoplasm in favor of CML (myeloblast 25%). Immuno-phenotyping on bone marrow aspiration showed that ~22% of the peripheral blood total cells were myeloblasts because of the positivity for CD117, CD34, HLA-DR, CD33, CD13, and Cyto MPO, this case appears to be a CML in the transformation phase to acute myeloid leukemia (CD34/CD117) (Table 2).

**Table 2:** Immuno-phenotyping on bone marrow aspiration results before and after treatment.

CD13	CD117	CD33	MPO	HLA-DR
66%	22%	70%	25%	24%
		After 4 weeks		
20%	3.73%	18%	4%	20%

**Figure 3:**
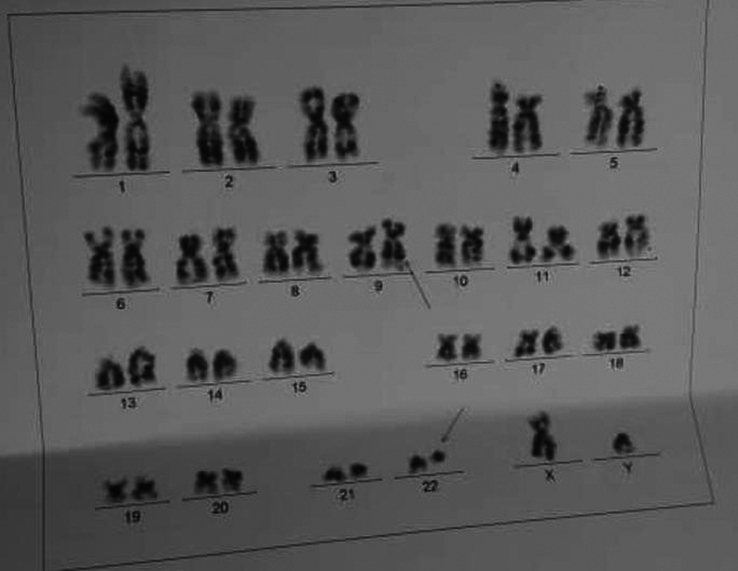
BCR-ABL flow cytometry fish on bone marrow sample was 100% positive.

The bone marrow biopsy showed markedly hypercellular bone marrow (100%) due to the proliferation of myeloid precedors with mild left shift and no significant increase in blasts, and the eosinophil and plasma cell counts were normal. The number of erythroid series is markedly decreased, the number of megakaryocytes is mildly increased, and megakaryocytes are small, with no fibrosis or necrosis. BCR-ABL flow cytometry fish on bone marrow sample was 100% positive (Fig. 3).

According to all these investigations, the patient was diagnosed with CML transformed to AML, and the patient was treated with imatinib 400 mg once daily, cytarabine 150 mg/m^2^ for 7 days, and daunorubicin75 mg/m^2^ for 3 days as induction chemotherapy (7+3)^6,7^.

Because of the leukostasis, we added hydroxyurea 1 g twice daily, and allopurinol 300 mg once daily.

A bone marrow aspiration for evaluation was performed after 4 weeks revealing a positive BCR-ABL fusion gene at 80%, and the flow cytometry showed minimal residual disease of 3.73% means a partial response did not complete response (Table 2).

A consultation was made with the bone marrow transplant center, and a dose of [Ida-flag] (idarubicin, fludarabine, high dosage cytarabine, and granulocyte colony-stimulating factor G-CSF) was added to imatinib to prepare the patient for transplation.

The dosage of FLAG-IDA we used was (G-CSF from the first day to the seventh 300 mg every day, from the second day to the fourth Idarubicin 16 mg [10 mg/m^2^], from the second day to the sixth cytarabine 2.4 g [1.5 g/m^2^] then Fludarabine 50 mg [30 mg/m^2^])^6,7^.

Supportive care was started by prophylaxis and management of tumor lysis syndrome, infection, hyperfibrinolysis, bleeding, and eventual thrombosis with allopurinol, acyclovir, nystatin, and prednisolone as eye drops.

The follow-up visit, after 4 weeks the patient had a partial recovery with clinical improvement, and a bone marrow aspiration was performed for evaluation, and the BCR-ABL gene ratio was 1.58%, which meant that the patient had a complete remission.

## Discussion

CML is the most common leukemia in adults and counts 20% of leukemias, it affects the peripheral blood and the bone marrow, with unknown risk factors^8^. About 90–95% of patients with CML have a shortened chromosome 22 because of a reciprocal translocation t (9; 22) (q34; q11.2) known as the Philadelphia chromosome (BCR-ABL1 fusion gene)^9^. CML can have a triphasic clinical course based on the percentage of immature white blood cells (blasts) found in the blood and bone marrow, the three phases are chronic, accelerated, and blast phase. In CML the number of blasts is higher than 5%, but usually less than 10%. Fifteen percent or more blasts is a sign of advanced phase CML (accelerated and blast phase). Our patient was diagnosed with an advanced phase of CML^8^. A blast phasis is defined as the presence of greater than 20% blasts in the peripheral blood or bone marrow. The blasts could be characterized as either myeloid (60–80% of cases) resulting in acute myeloblastic leukemia, or lymphoid. Our patient had ~22% myeloid blast on his bone marrow so the diagnosis was CML in blast phase with AML^10^. Approximately half of the patients with CML are asymptomatic, and most patients are diagnosed in the chronic phase of CML.CML often presents with symptomatic anemia such as fatigue malaise, and splenomegaly with left upper quadrant fullness, or pain. As CML progresses into the accelerated phase or blast phase, symptoms such as headaches, bone pain, fever, joint pain, and bleeding become more common, like our patient^8^. Studies have shown that only 5% of CML patients present with ocular symptoms at the initial diagnosis^11^. Ocular involvement in leukemia can precede the diagnosis of leukemia or can occur during the disease course itself^12^. Ocular complications of leukemia are known to be due to direct infiltration of the ocular tissues or as a result of vascular abnormalities, prolonged leukocytosis in CML that causes increased blood viscosity. Leukemic retinopathy is characterized by the presence of multiple pre-retinal, intraretinal, or subretinal hemorrhages, other clinical signs include Roth’s spots^13^. Our patient had bilateral retinal blot aneurysm hemorrhages and hemorrhage over both foveae.

The treatment of choice in CML transforming to AML is induction chemotherapy, besides TKI tyrosine kinase inhibitors like imatinib^6,7^. We gave our patient induction standard 7+3 (cytarabine, and daunorubicin) with imatinib. Allogenic hematopoietic stem cell transplantation (allo-HSCT) is an important consideration for resistant chronic phase and advanced phases of CML, and represents a pivotally important treatment strategy in fit adults with AML^14^. after at least two courses of intensive induction therapy, roughly 20% of younger AML patients do not achieve complete remission (CR), and 70% of patients who obtain CR will relapse^15^. for these considerations we sent our patient to a center specialized in marrow transplantation, after he treated with FLAG-IDA we used was (G-CSF, idarubicin, cytarabine, and fludarabine)^6,7^.

Ophthalmic manifestations are considered a rare presentation of CML, only found in the literature as case reports or small case series^16^. our patient was CML in the blast phase with AML.

Yassin and colleagues described a review in 2022 they found 40 cases of CML with ocular manifestations, most of them were initial presentations of CML except three, and only five of them were in relapses, with retinal hemorrhages as the most common finding. The remaining three cases were CML progression to the blast phase. In our case the patient had retinal hemorrhages as the first sign of CML but in the blast phase^17^.

The challenges in our case are that the patient presented with nonspecific ocular manifestation at the initial diagnosis of CML, which transformed into acute myeloid leukemia immediately, in addition, we could not study the other mutation analysis tests because they are not available in our country.

Fortunately, the patient had a matching donor with his sibling, so we prepared him for transplantation at the first CR.

## Conclusion

It is important to recognize early clinical changes in patients who do not present with the usual signs and symptoms of CML. However, the ocular manifestations of CML are unique and nonspecific and may be the first presenting symptom of CML. An eye problem requires an urgent evaluation so we should take it into consideration to obtain the right diagnosis and better treatment. Close coordination between hematologists and ophthalmologists is important.

## Ethical approval

Ethical approval by Ethical committee of Faculty of medicine, Damascus University, Syrian Arab Republic.

## Consent

Written informed consent was obtained from the patient’s parents/legal guardian for publication and any accompanying images Institutional approval is not required for this case study.

## Source of funding

Not applicable.

## Author contribution

Y.A. concepted, designed and acquired data for the work and analyzed it. S.S. shared his expert opinion to support treatment decision and also revised it.

## Conflicts of interest disclosure

The authors declare no conflicts of interest.

## Research registration unique identifying number (UIN)

1. Name of the registry: Researchregistry.com.

2. Unique Identifying number or registration ID: researchregistry 10276.

3. Hyperlink to your specific registration: https://www.researchregistry.com/browse-theregistry#home/?view_2_search=10276&view_2_page=1.

## Guarantor

Yara Al Ammouri.

## Data availability statement

The case data are available.

## Provenance and peer review

Not commissioned, externally peer-reviewed.
